# Age at menarche is inversely related to the prevalence of uterine cancer

**DOI:** 10.1186/s40001-025-02472-z

**Published:** 2025-03-26

**Authors:** Ning Chen, Xiaohui Pei, Hao Sun, Yaoyun Zhang, Mengmeng Wang, Ziqian Song, Jialin Wang, Yuantao Qi

**Affiliations:** 1 Shandong Cancer Hospital and Institute, Shandong First Medical University and Shandong Academy of Medical Sciences, Jinan, 250117 China; 2 The Second Affiliated Hospital, Shandong First Medical University and Shandong Academy of Medical Sciences, Taian, 271000 Shandong China; 3 Xiyuan Hospital, Chinese Academy of Traditional Chinese Medicine, Beijing, 100091 China; 4 Binhai County People’s Hospital, Yancheng, 224500 Jiangsu China

**Keywords:** Menarche, Uterus, Ovaries, NHANES

## Abstract

**Objectives:**

The objective of this study was to investigate the relationship between the age of menarche and the prevalence of malignancies of the uterus and ovaries.

**Methods:**

A total of 5540 women were screened from those who participated in the National Health And Nutrition Examination Survey (NHANES) questionnaire from 2007 to 2020, and their variable factors of age, race, education level, Poverty Impact Ratio (PIR), marital status, Body Mass Index (BMI), waist circumference, duration of moderate exercise, smoking habits, hypertension status, energy intake, diabetes and alcohol consumption habits were analysed statistically and by logistic regression.

**Results:**

Univariate and multivariate logistic regression analysis of the relationship between age at menarche and gynaecological cancer (uterus/cervix/ovary cancer, the following gynecologic cancers in the article refer to having at least one of these three cancers) prevalence showed a negative association between age at menarche and malignancies of the uterus and ovaries prevalence (OR: 0.82, 95% CI 0.69–0.97), with a statistically significant difference (*p* = 0.02). Regression results of the association between age at menarche and different types of malignancies of the uterus and ovaries found a negative association between age at menarche and prevalence in uterine cancers (*P* = 0.03) and no association between age at menarche and prevalence in cervical and ovarian cancers (*P* = 0.17, *P* = 0.29). Those with a younger age at menarche were more likely to develop uterine cancer (OR: 0.72, 95% CI 0.54–0.98).

**Conclusions:**

There was a correlation between age at menarche and malignancies of the uterus and ovaries, with those who had menarche at an earlier age being at a higher risk of uterine cancer.

## Introduction

Gynecological cancer was a cancer with high morbidity and mortality rates, and it was the most common cancer among women [1, 2]. Gynecological cancers posed a great threat to the physical and psychological health of women worldwide, and even caused significant economic pressure on societies and countries [3, 4]. The most common gynecologic cancer is cancer of cervix, followed by ovary and uterus [5]. Each of these cancers was unique in type, with different curative factors, risk factors, diagnosis and treatment, and prognostic outcomes [6]. However, through the control of risk factors, early screening of related factors, screening of high-risk groups and other means, reducing risk of the occurrence of gynecological cancer [1, 7].

When a woman's hypothalamus–pituitary–gonad axis tended to mature, various parts of the woman's body would change, such as breast development and increased body hair. However, menarche was the most important manifestation [8]. There were many factors affecting the age of menarche, such as hormone level [9], family economic condition [10, 11], birth weight [12] and so on. In addition, the age of menarche played a certain role in the occurrence of many diseases. A study on the relationship between age at menarche and cardiac function showed that there was a negative correlation between them [13]. The age of menarche had also been proved to be related to respiratory diseases [14], multiple sclerosis [15], myopia [16], cardiovascular disease prognosis [17] and so on. As an important factor of women's physical index, the relationship between the age of menarche and gynecological cancer had aroused our interest. The important thing was that the relationship between the age of menarche and gynecological cancer was not clear.

To investigate the relationship between age at menarche and gynaecological cancer (uterus/cervix/ovary cancer, the following gynecologic cancers in the article refer to having at least one of these three cancers) prevalence, we conducted a statistical analysis of questionnaire data from the National Health and Nutrition Examination Survey (NHANES). The NHANES database was used as the data source for all data in this paper. In this study, a total of 13 factors that may have an impact on the results were considered and adjusted accordingly. These included a number of dimensions, such as basic demographic information, personal fitness, lifestyle habits and disease status, with a number of factors such as age, marriage, body mass index and income having a significant effect on cancer prevalence. Several sensitivity analyses, subgroup analyses, and cross-validation were conducted to ensure the reliability and accuracy of the results based on these covariates that may have an impact on the results. In this study, we focused on the most common cancers among gynaecological cancers. We screened data from NHANES questionnaires from 2007 to 2020 and analyzed the data statistically. The relationship between age at menarche and the prevalence of gynecologic cancers was ultimately assessed.

## Materials and methods

### Study design and sample

**Fig. 1:**
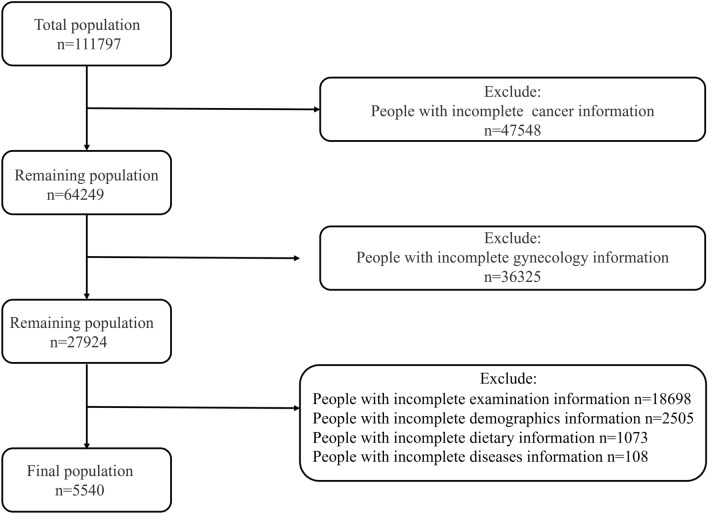
Screening flow chart of the study population

NHANES covers the areas of demographics, health status, lifestyle, and healthcare utilization and is designed to assess the health and nutritional status of the U.S. population. These data, which are nationally representative using stratified random sampling and ensure validity through rigorous quality control, are widely used in public health research and policy development. The cancer-related data used in this study were mainly derived from the questionnaire interview data therein, collected through household interviews and computer-assisted questionnaires. The section on reproductive health is a database of variables routinely collected primarily for females 12 years of age and older. These questions are administered at the Mobile Examination Center (MEC) by trained interviewers through the Computer Assisted Personal Interview (CAPI) system, which supports proxy access and interpretation services. The CAPI system has built-in consistency checks and help functions to ensure data quality and minimize input errors. Health and nutrition data for the study population were obtained from the NHANES database. Some specific information on this survey is described elsewhere [18, 19]. All the data of NHANES 2007–2020 were approved by the Ethics Review Committee, and all subjects had informed consent and written proof. The number of population initially enrolled in the study was 111,797, and 47,548 were excluded from the study because of incomplete cancer information. In addition, we excluded 36,325 subjects with incomplete gynecological information. Of the remaining 27,924 study subjects, we further excluded a number of study subjects. These included study subjects with missing information on examination (*n* = 18,698), missing information on demographics (*n* = 2505), missing information on diet (*n* = 1073), and missing information on disease (hypertension and diabetes) (*n* = 108). A total of 5540 subjects were eligible for the experimental study. The screening process for the study population is shown in Fig. 1.

### Assessments

Population screening criteria were age > 18 years, complete and reliable data in all categories, and clear representation weights. The definition of the type of cancer was assessed according to the location of the cancer and the doctor's diagnosis. The statistics of the age of menarche were based on the results of a questionnaire. Considering that the study was on a female population, marital status was considered an important reference variable factor. Marital status was classified as: 1. married; 2. never married; and 3. widowed/divorced/separated. Some other relevant characteristics about the study population were based on the NHANES findings. Data were obtained on age (< 65/ ≥ 65), race (Non-Hispanic White, Non-Hispanic Black, Mexican American, Other Hispanic, and other race), education level (less than high school, high school, and more than high school). The poverty impact ratio (PIR) was used as a threshold for the variable at 2.8%. In addition, the inclusion criteria of waist circumference (< 94 cm/ ≥ 94 cm), body mass index (BMI) (< 25 kg/m^2^/ ≥ 25 kg/m^2^), moderate exercise (< 45 min/ ≥ 45 min) and energy intake (< 1700 kcal/ ≥ 1700 kcal) were also evaluated according to the data in the questionnaire. Where the energy intake value was calculated as an average value based on the total energy intake over 2 days, the daily energy intake is derived from the total energy from food and beverages. Moderate exercise was defined as moderate intensity exercise, fitness or recreational activity that results in a small increase in breathing or heart rate and was performed continuously for at least 10 min.

The study assessed participants' moderate exercise duration (< 45 min/ ≥ 45 min) and energy intake (< 1700 kcal/ ≥ 1700 kcal). Moderate exercise is defined as moderate-intensity sports, fitness, or recreational activities that result in a slight increase in breathing or heart rate and are performed continuously for at least 10 min. “Moderate exercise” obtained data primarily through a questionnaire approach, based on the Global Physical Activity Questionnaire (GPAQ), with respondent-level physical activity interview data. “Energy intake” data were derived from the sum of energy consumed in food and beverages in the 24 h prior to the interview for NHANES participants. All participants participate in two 24-h dietary session interviews, the first dietary recall interview collected in person at the Mobile Examination Center (MEC), and the second interview collected by telephone 3–10 days later. The standards for calculating energy in foods and beverages come from the USDA's Food and Nutrition Database for Dietary Studies (FNDDS), which is produced by the USDA's Nutrition Data Laboratory. Energy intake is calculated as an average based on the total energy intake for 2 days, whereas daily energy intake is derived from the total energy from food and beverages.

The smoking habits of the study population were assessed into the following three categories: 1 nexer (smoked less than 100 cigarettes in life); 2 former (smoked more than 100 cigarettes in life and smoke not at all now); and 3 now (smoked more than 100 cigarettes in life and smoke some days or every day). The criteria for classifying drinking habits were based on the most recent assessment indicators (only female criteria have been used, as well as criteria for the shared component): 1 never (had < 12 drinks in lifetime); 2 former (had ≥ 12 drinks in 1 year and did not drink last year, or did not drink last year but drank ≥ 12 drinks in lifetime); 3 mild (≤ 1 drink per month); 4 moderate (≤ 2 drinks per month); and 5 heavy (≥ 3 drinks per month).

The diagnosis of the underlying disease reported in the investigation was assessed on the basis of the results of the examination and the doctor's diagnosis. Among them, the diagnostic criteria of hypertension: average blood pressure ≥ 140/90 mmHg. Average blood pressure was calculated by the following protocol: 1 the diastolic reading with zero was not used to calculate the diastolic average; 2 if all diastolic reading were zero, then the average would be zero; 3 if only one blood pressure reading was obtained that reading was the average; and 4 if there was more than one blood pressure reading, the first reading was always exclude from the average. With regard to the inclusion criteria for diabetes, the assessment was classified according to the international customary criteria and related indicators: 1 doctor told you have diabetes; 2 glycohemoglobin HbA1c (%) > 6.5; 3 fasting glucose (mmol/L) ≥ 7.0; 4 random blood glucose (mmol/L) ≥ 11.1; 5 2-h OGTT blood glucose (mmol/L) ≥ 11.1; and 6 use of diabetes medication or insulin.

### Statistical analyses

For random missing samples, we used multiple interpolation to fill in. For non-random missing data, we excluded them and the filtered data were analysed using R (v.4.2.1). For continuous variables, we used mean and 95% confidence intervals (x ® (95%CI) for statistical descriptions, and *t* tests and ANOVA for between-group comparisons. For categorical variables, we used *p* values and 95% CI for statistical descriptions and chi-square tests for comparisons between groups. For the adjustment analyses, we chose binary logistic regression for multifactor analysis and multiple sensitivity analysis. Twelve covariates were stratified into the model to ensure the reliability of the analyses. In addition, we further fitted separate models for the association between age at menarche and the prevalence of three common gynaecological cancers. Several subgroup analyses and interaction studies were also performed to obtain a more precise target population. We used three section restricted cubic spline (RCS) to explore the nonlinear relationship between menarche age and the prevalence of common gynecological cancers and uterine diseases. In this analysis, adjustments were made for all 12 covariates. In this study, *P* < 0.05 was considered statistically significant difference. The NHANES complex multi-stage sampling design was used for all analyses in this study. Appropriate weights were selected for calculations and weighted multivariate logistic regression to ensure a representative sample. In addition, we conducted several sensitivity analyses to verify the reliability of the results. We constructed multiple models adjusting for age, race, education level, marital status, BMI, PIR, waist circumference, moderate activity time, smoking, alcohol consumption, hypertension, and diabetes.

## Results

This study included 5,540 NHANES participants, which could represent 38,016,087 US women, with an overall weighted prevalence of 1.91% for gynaecological cancers. The baseline characteristics of the study population are shown in Table 1. This study reveals that patients with malignant uterine and ovarian tumors differ significantly from non-patients on several characteristics. The patients were on average older (*P* < 0.01), had a lower level of education, with significantly fewer completing high school and beyond (*P* < 0.01), and had a lower (PIR) (*P* = 0.03), suggesting that socioeconomic status may have an impact on tumor risk. In terms of health and metabolic characteristics, patients had significantly higher BMI and waist circumference than nonpatients (*P* < 0.01) and a higher prevalence of hypertension (*P* < 0.01), suggesting that metabolic abnormalities may be important risk factors. In addition, the distribution of smoking behavior of patients was significantly different from that of non-patients. The percentage of current smokers was as high as 32.85%, which was significantly higher than 11.70% in non-patients (*P* < 0.01). The age of menarche, on the other hand, was slightly later than that of non-patients (*P* = 0.02). Although drinking behavior and proportion of diabetes mellitus did not show significant differences, the proportion of patients with heavy drinking and diabetes mellitus was relatively high. Overall, these results suggest that age, socioeconomic status, lifestyle and metabolic health have a significant impact on the development of malignant uterine and ovarian tumors.

**Table 1:** Baseline characteristics of the study population

Characteristics	Malignancies of the Uterus and Ovaries		*p* value
	No	Yes	
Total	98.09	1.91	
Age ~ years	46.74(46.05,47.42)	51.25(48.42,54.07)	< 0.01
Race ~ %			0.25
Non-Hispanic White	74.47(72.06,76.88)	73.05(63.16,82.94)	
Non-Hispanic Black	9.10(7.82,10.38)	5.59(2.05, 9.13)	
Mexican American	5.47(4.46,6.48)	4.11(1.66,6.56)	
Other Hispanic	4.39(3.64, 5.13)	6.84(2.44,11.23)	
Other Race	6.56(5.71, 7.41)	10.41(2.50,18.32)	
Education level ~ %			< 0.01
Less than high school	6.30(5.44, 7.16)	15.54(7.67,23.40)	
High school	17.72(16.29,19.15)	18.44(11.50,25.38)	
More than high school	75.98(74.19,77.78)	66.03(55.51,76.55)	
Marital Status			0.01
Married	63.01(61.06,64.96)	63.76(54.26,73.26)	
Never married	17.10(15.51,18.70)	6.03( 2.08, 9.99)	
Widowed/Divorced/Separated	19.89(18.62,21.15)	30.21(21.27,39.14)	
Family PIR ~ %	3.38(3.30,3.46)	2.95(2.57,3.33)	0.03
BMI ~ kg/m^2^	28.27(28.00,28.54)	30.52(28.67,32.37)	0.01
Waist ~ cm	94.53(93.92, 95.15)	100.87(97.06,104.68)	< 0.01
Moderate exercise ~ minutes	53.29(51.86,54.72)	63.92(50.01,77.84)	0.04
Smoking behavior ~ %			< 0.01
Never	66.02(64.03,68.00)	37.51(24.50,50.51)	
Former	22.29(20.59,23.98)	29.64(17.42,41.86)	
Now	11.70(10.57,12.82)	32.85(21.72,43.99)	
Alcohol consumption ~ %			0.55
Never	10.96(9.47,12.45)	7.03(2.39,11.67)	
Former	8.06(6.95, 9.17)	9.44(3.23,15.65)	
Mild	36.61(34.49,38.74)	36.77(24.03,49.50)	
Moderate	26.35(24.77,27.92)	26.12(14.27,37.97)	
Heavy	18.02(16.53,19.50)	20.64(11.56,29.73)	
Hypertension ~ %			< 0.01
Yes	29.22(27.50,30.93)	48.49(36.87,60.11)	
No	70.78(69.07,72.50)	51.51(39.89,63.13)	
Diabetes ~ %			0.12
Yes	9.64(8.78,10.51)	16.39(6.38,26.40)	
No	90.36(89.49,91.22)	83.61(73.60,93.62)	
First menstruation ~ years	12.20(11.78,12.62)	12.72(12.66,12.77)	0.02
Year cycle ~ %			0.16
2007–2008	13.26(11.05,14.48)	14.43(6.70,22.16)	
2009–2010	13.34(11.76,14.91)	16.69(8.64,24.74)	
2011–2012	15.46(13.14,17.78)	10.47(0.25,20.69)	
2013–2014	15.11(11.86,31.45)	21.65(11.86,31.45)	
2015–2016	15.59(13.01,30.39)	21.84(13.30,30.39)	
2017–2018	13.58(11.78,15.38)	7.05(2.19,11.92)	
2019–2020	13.65(11.92,15.38)	7.86(3.42,12.30)	

OR odds ratio, CI confidence interval, PIR property income ratio, BMI body mass index. Data are shown as mean (x ®) or n (%), combined with 95% CI

The results of the univariate and multivariate logistic regression analyses between age at menarche and malignancies of the uterus and ovaries prevalence are shown in Table 2. Univariate logistic regression analysis showed a negative association between age at menarche and malignancies of the uterus and ovaries prevalence (OR: 0.82, 95% CI 0.69–0.97), with a statistically significant difference (*P* = 0.02). Model 1 was adjusted for age and ethnicity and showed a negative association between age at menarche and malignancies of the uterus and ovaries prevalence (OR: 0.81, 95% CI 0.68–0.96), with a statistically significant difference (*P* = 0.02). Model 2 was adjusted for age, race, education level, PIR, marital status, BMI, waist circumference and duration of moderate exercise. The results showed a negative association between age at menarche and malignancies of the uterus and ovaries prevalence (OR: 0.82, 95% CI 0.69–0.98), with a statistically significant difference (*P* = 0.03). Model 3 was adjusted for age, race, education level, PIR, marital status, BMI, waist circumference, duration of moderate exercise, smoking habits, alcohol consumption habits, hypertension status and diabetes. The results showed a negative association between age at menarche and malignancies of the uterus and ovaries prevalence (OR: 0.84, 95% CI 0.71–1.00), with a statistically significant difference (*P* = 0.05). In all three sensitivity analyses, age at menarche was significantly and negatively associated with the prevalence of malignancies of the uterus and ovaries. Even in model 3, where we adjusted for a total of 12 covariates, the results were still significantly different. This suggested that age at menarche was stably associated with the prevalence of malignancies of the uterus and ovaries in all cases. It was worth noting that as the number of covariates adjusted increases, the effect of age at menarche on the prevalence of malignancies of the uterus and ovaries in women decreases.

**Table 2:** Univariate and multivariate logistic regression analyses between the age of menarche and the prevalence of malignancies of the uterus and ovaries

Outcomes	model	OR (95%CI)	*p* value
Malignancies of the Uterus and Ovaries	Crude	0.82(0.69,0.97)	0.02
	Model1	0.81(0.68,0.96)	0.02
	Model2	0.82(0.69,0.98)	0.03
	Model3	0.84(0.71,1.00)	0.05

**Crude is an unadjusted model, Model1 is a model adjusted for age and race, Model2 is a model adjusted for age, race, education level, PIR, marital status, BMI, waist circumference and moderate exercise time, and Model3 is a model adjusted for age, race, education level, PIR, marital status, BMI, waist circumference, moderate exercise time, smoking habits, alcohol consumption, hypertension status, and diabetes

**Fig. 2:**
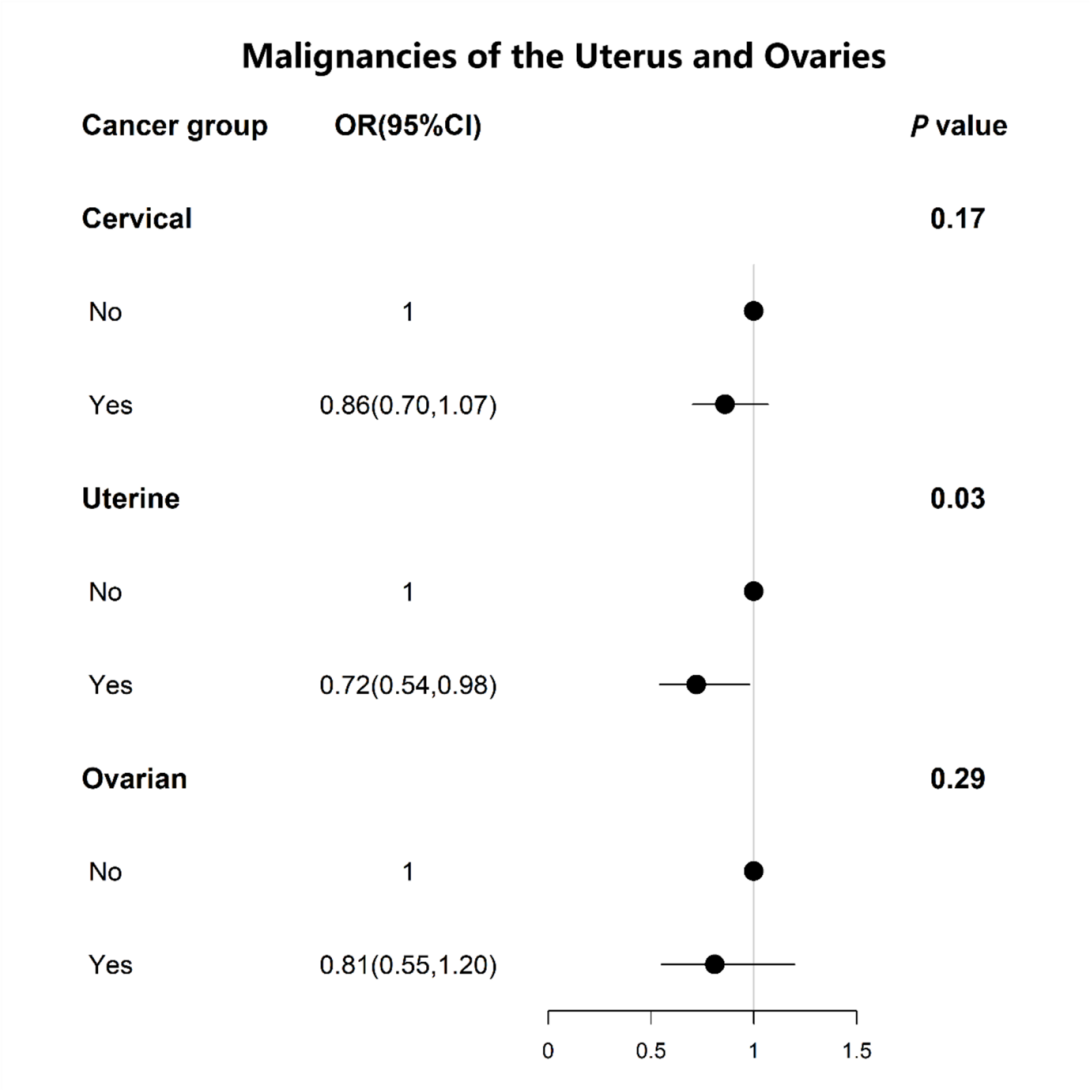
Results of regression analysis between the age of menarche and the prevalence of malignancies of the uterus and ovaries were analyzed after adjustment according to the basic variables

**Fig. 3:**
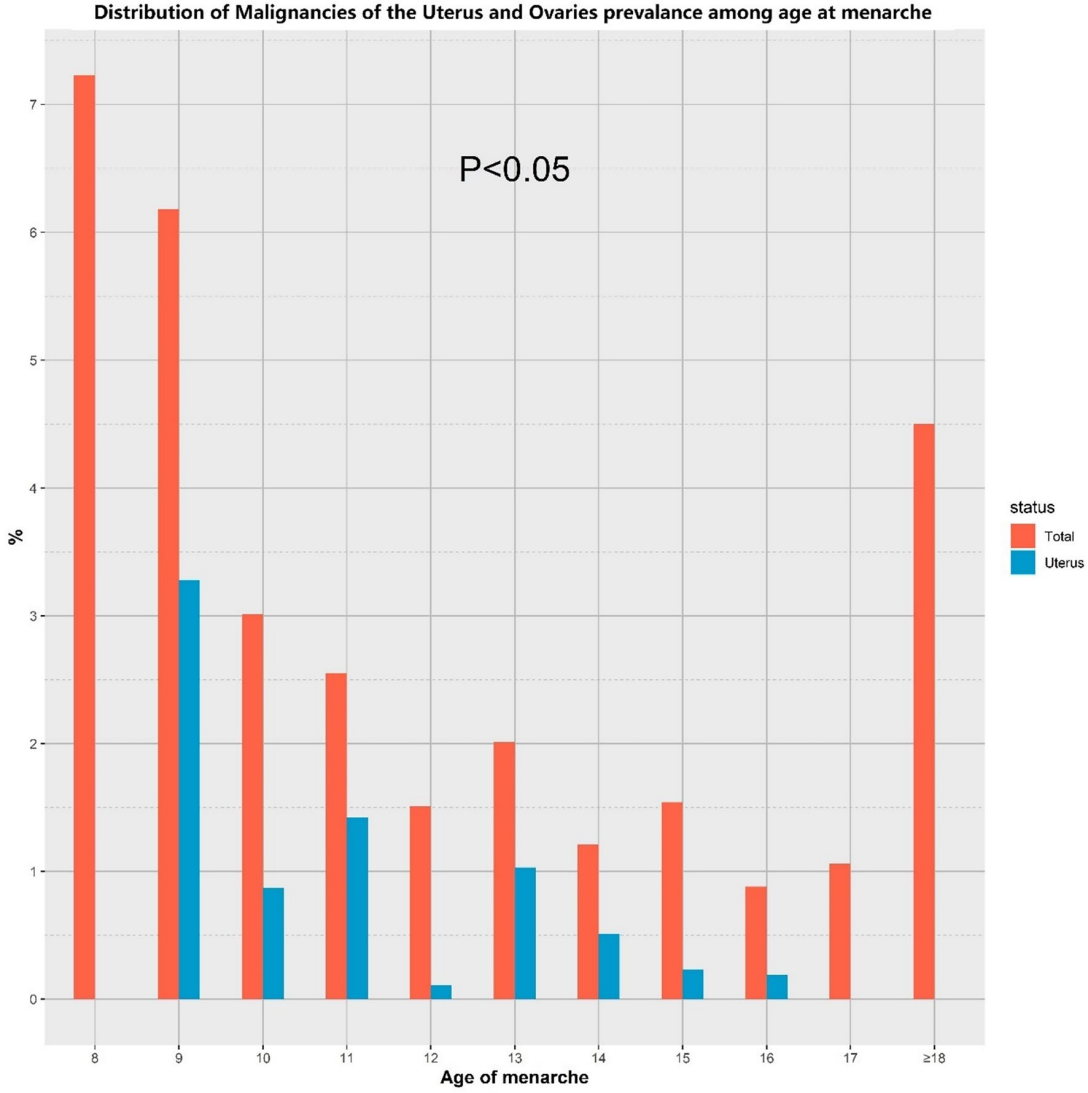
Prevalence of malignancies of the uterus and ovaries in women of different menarcheal ages and trends in the distribution of malignancies of the uterus and ovaries

**Fig. 4:**
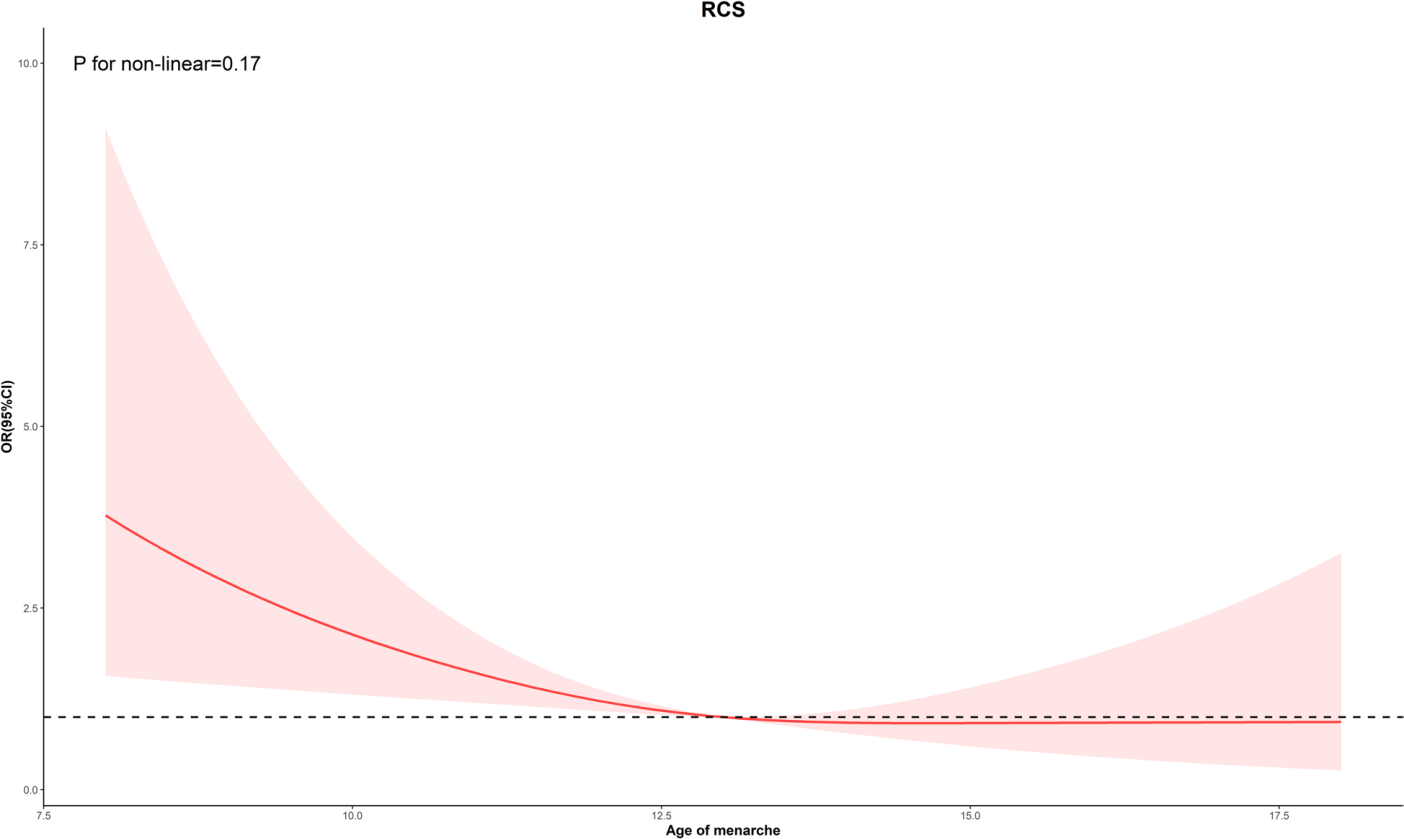
Restricted cubic spline (RCS) Showing age of menarche and malignancies of the uterus and ovaries prevalence

**Fig. 5:**
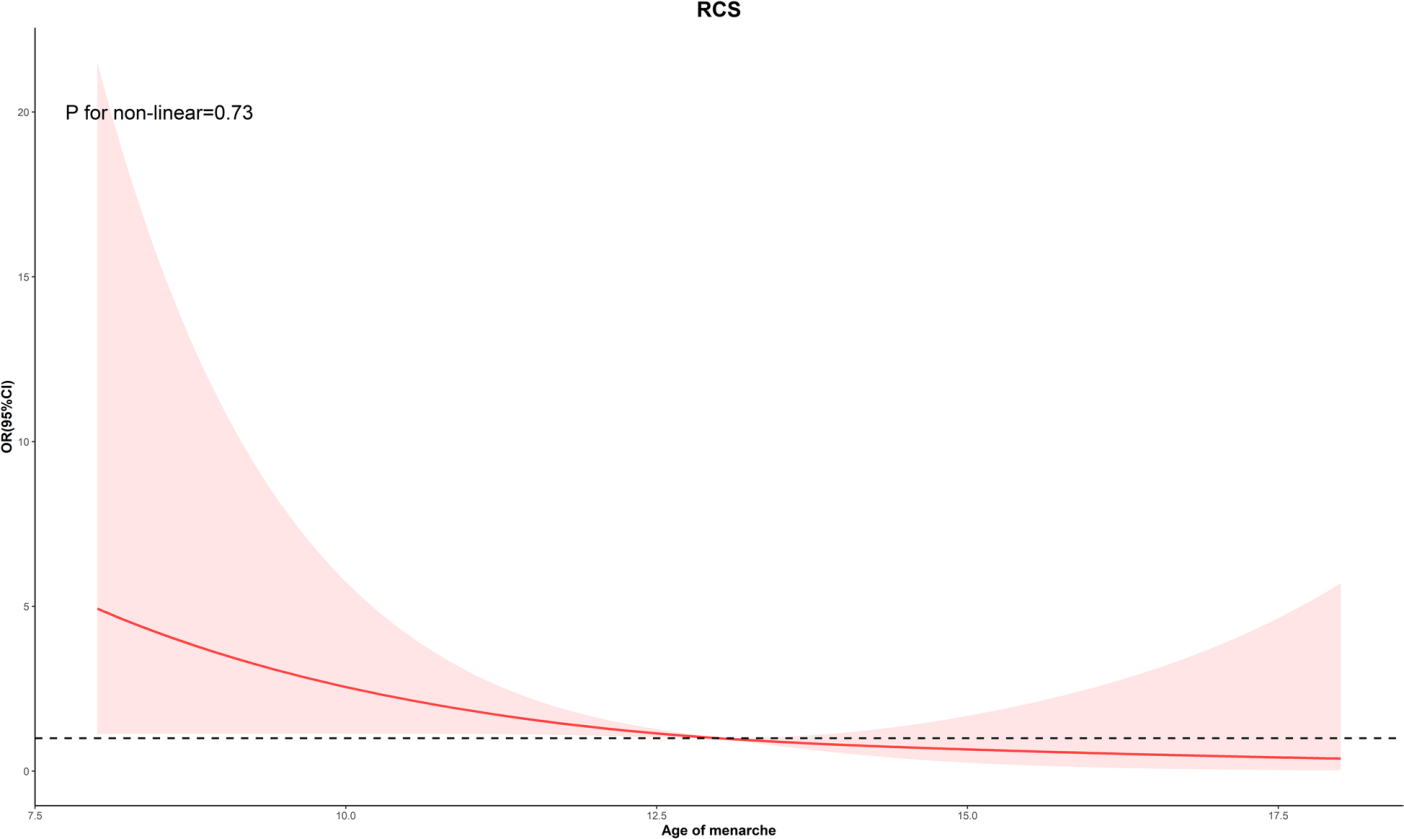
Restricted cubic spline (RCS) Showing age of menarche and uterine cancer prevalence

As shown in Fig. 2**,** the relationship between age at menarche and the prevalence of malignancies of the uterus and ovaries. We conducted regression analyses adjusted for basic demographic factors (age, race, education level, PIR, marital status). The results found a negative correlation between age at menarche and prevalence in uterine cancer (*P* = 0.03). No correlation between age at menarche and prevalence in cervical cancer (*P* = 0.17) and ovarian cancer (*P* = 0.29). Population with a young age of menarche were more likely to develop uterine cancer (OR: 0.72, 95% CI 0.54–0.98). In addition, trends in the prevalence of malignancies of the uterus and ovaries and the distribution of uterine cancers among malignancies of the uterus and ovaries by age at menarche were studied according to the age of menarche (Fig. 3). The findings showed that there was an overall downward trend in the prevalence of gynaecological and uterine cancers as the age of menarche increases until the age of 18. The results of the non-linear analysis between age of menarche and malignancies of the uterus and ovaries prevalence, as shown in Fig. 4, revealed a significant negative linear relationship (P non-linear = 0.17). This relationship also appears in the prevalence of uterine cancer (P non-linear = 0.73) (Fig. 5).

The results of the subgroup analysis are shown in Table 3. No significant interactions with the albumin/globulin ratio were found for any of the subgroup variables. However, earlier age at menarche was associated with a greater risk of cancer among those with high school education or higher (*P* = 0.05), unmarried (*P* < 0.01), small BMI (*P* = 0.02), long duration of daily physical activity (*P* = 0.02), moderate alcohol consumption (*P* = 0.01), and no diabetes mellitus (*P* = 0.03). The results of the subgroup analysis on uterine cancer are shown in Table 4**.** Age at menarche increased the risk of uterine cancer in several subgroups, but only waist circumference and hypertension interacted with age at menarche. However, it did not show a significant association with the risk of uterine cancer in either of the two subgroups of waist circumference levels, whereas in those with hypertension. The younger the age at menarche, the higher the risk of uterine cancer (OR: 0.63, 95%CI 0.40–0.99).

**Table 3:** Results of subgroup analysis related to malignancies of the uterus and ovaries

Subgroup variable	Malignancies of the uterus and ovaries		
	OR (95%CI)	*p* value	*P* for interaction
Age			0.47
< 65	0.88(0.73,1.06)	0.17	
≥ 65	0.72(0.51, 1.02)	0.06	
Race			0.52
Non-Hispanic White	0.82(0.64,1.05)	0.11	
Non-Hispanic Black	0.87(0.65, 1.16)	0.34	
Mexican American	1.46(0.96, 2.22)	0.08	
Other Hispanic	0.70(0.30, 1.66)	0.42	
Other Race	0.64(0.36,1.13)	0.12	
Education level			0.72
Less than high school	0.77(0.51, 1.15)	0.20	
High school	0.80(0.58, 1.11)	0.17	
More than high school	0.81(0.66, 1.00)	0.05	
Marital Status			0.50
Married/Living with partner	0.83(0.67, 1.02)	0.08	
Never married	0.56(0.38,0.82)	< 0.01	
Widowed/Divorced/Separated	0.87(0.63, 1.19)	0.38	
Family PIR			0.80
< 2.8	0.80(0.63, 1.02)	0.08	
≥ 2.8	0.88(0.70, 1.11)	0.28	
Waist			0.50
< 94	0.76(0.55, 1.04)	0.09	
≥ 94	0.88(0.72,1.08)	0.23	
BMI			0.21
< 25	0.67(0.48, 0.93)	0.02	
≥ 25	0.89(0.72,1.09)	0.26	
Moderate exercise			0.55
< 45	0.89(0.68, 1.17)	0.39	
≥ 45	0.81(0.67, 0.97)	0.02	
Smoking behavior			0.42
Never	0.93(0.75,1.16)	0.53	
Former	0.76(0.57, 1.02)	0.07	
Now	0.85(0.61, 1.18)	0.32	
Alcohol consumption			0.22
Never	0.79(0.51,1.24)	0.31	
Former	1.14(0.81,1.61)	0.43	
Mild	0.80(0.57,1.11)	0.18	
Moderate	0.72(0.57,0.90)	0.01	
Heavy	0.94(0.63,1.41)	0.77	
Hypertension			0.37
Yes	0.79(0.60,1.04)	0.10	
No	0.88(0.72, 1.07)	0.20	
Diabetes			0.44
Yes	0.89(0.62, 1.27)	0.52	
No	0.81(0.67, 0.98)	0.03	

*Models were adjusted for age, race, education level, PIR, marital status, BMI, waist circumference, moderate exercise time, smoking habits, alcohol consumption, hypertension status, and diabetes. (except subgroup variables); PIR, energy intake, waist circumference, and duration of moderate exercise were divided into groups based on median

**Table 4:** Results of subgroup analysis related to uterine cancer

Subgroup Variable	Cancer = Uterine		
	OR (95% CI)	*p* value	*P* for interaction
Age			0.37
< 65	0.88(0.63, 1.22)	0.43	
≥ 65	0.62(0.39, 0.97)	0.04	
Race			0.20
Non-Hispanic White	0.77(0.50,1.18)	0.23	
Non-Hispanic Black	0.46(0.21,0.98)	0.05	
Mexican American	1.65(0.94,2.89)	0.08	
Other Hispanic	0.10(0.01,1.09)	0.06	
Other Race	0.83(0.66,1.05)	0.11	
Education level			0.10
Less than high school	0.57(0.31, 1.05)	0.07	
High school	0.65(0.20,2.10)	0.47	
More than high school	0.69(0.47, 1.00)	0.05	
Marital Status			0.32
Married/Living with partner	0.80(0.60, 1.06)	0.12	
Never married	3.54(2.24, 5.58)	0.02	
Widowed/Divorced/Separated	0.59(0.37, 0.92)	< 0.01	
Family PIR			0.23
< 2.8	0.58(0.37, 0.90)	0.02	
≥ 2.8	0.91(0.61, 1.36)	0.65	
Waist			0.01
< 94	1.29(0.99,1.69)	0.06	
≥ 94	0.69(0.46, 1.04)	0.08	
BMI			0.39
< 25	0.94(0.58,1.53)	0.81	
≥ 25	0.73(0.49, 1.07)	0.11	
Moderate exercise			0.79
< 45	0.70(0.39, 1.27)	0.24	
≥ 45	0.77(0.62, 0.95)	0.02	
Smoking behavior			0.48
Never	0.79(0.58, 1.07)	0.12	
Former	0.69(0.39, 1.22)	0.20	
Now	0.83(0.50,1.36)	0.45	
Alcohol consumption			0.36
Never	0.37(0.12,1.16)	0.09	
Former	0.82(0.47, 1.43)	0.48	
Mild	0.69(0.40,1.16)	0.16	
Moderate	0.68(0.41, 1.12)	0.13	
Hypertension			0.04
Yes	0.63(0.40, 0.99)	0.05	
No	1.00(0.81, 1.24)	0.99	

*Models were adjusted for age, race, education level, PIR, marital status, BMI, waist circumference, moderate exercise time, smoking habits, alcohol consumption, hypertension status, and diabetes. (except subgroup variables); PIR, energy intake, waist circumference, and duration of moderate exercise were divided into groups based on median

## Discussion

Menarche was a very important event in a woman's life, which was affected by life, family, body and other factors. Menarche also played a role in the occurrence of diseases [8–17]. According to the statistical analysis and logical regression analysis of the questionnaire data of NHANES 2007–2020, the results showed that there was a correlation between the age of menarche and the prevalence of gynecological cancer. We conducted a series of analyses on age, race, education, PIR, marital status, BMI, waistline, moderate exercise time, smoking habits, hypertension, energy intake, diabetes and drinking habits.

Age was considered to be a highly correlated factor in cancer risk. At present, elderly patients account for the majority of all cancer patients, including cancer recurrence [20–22]. Patients who were younger at menarche and older at the time of the survey may experience more times of menarche, which also had a certain impact on the occurrence of gynecological cancer. During menstruation, a woman's body would be altered in terms of trace elements and body status [23]. The process of menstruation was accompanied by metabolic changes, and the relationship between the occurrence and development of cancer and metabolism was very close [24–27]. When a woman's menstrual cycle increases, it also indicates frequent changes in the body's metabolic state. The younger the age of menarche, the greater the change in estrogen levels and the progressively higher the risk of cancer [28].

Moreover, obesity and birth weight are factors that influence the age of menarche [29, 30]. An experiment by Moslshi et al. in 2021 found an association between intake of plant and animal protein in childhood and age at menarche [31]. Waist circumference, BMI, moderate exercise time, PIR, energy intake, smoking and alcohol consumption were all associated with obesity indices [32–34]. In a 2022 study on obesity among Japanese adolescents, a positive association between low household income and obesity was found [34]. In addition, many lifestyle habits played an important role in obesity in adolescents [35]. Obesity not only affected the age of first menstruation, but also played a role in the physical changes that occur after menarche. In this study, differences in the relationship between age at menarche and malignancies of the uterus and ovaries prevalence emerged in population of different weights. In the results of the subgroup analysis (Tables 3 and 4), the prevalence of uterine cancers was higher in those with a young age at menarche than in those with an older age at menarche among those with a normal weight. Similar statistical results were found for the other variable factors of interest (marital status, length of moderate exercise, alcohol consumption, and energy intake).

This study involved a variety of variable factors that are informative about the prevalence of malignancies of the uterus and ovaries. In addition, some of the variable factors had an impact on the age at menarche. More importantly, as age at menarche changed, age at menarche played a role in the variation of some of these variables. As a simple example, obesity itself was a factor that influences the onset of menstruation. After the onset of menstruation, obesity was a mediator between the onset of menstruation and the onset of disease. Multiple variable factors interacted and influenced each other, and the correlation between many of these variables needed to be verified by more experiments. Based on previous studies, it can be hypothesized that the older the age of first menstruation, the less estrogen exposure a woman has in her body, and thus the lower her risk of developing uterine cancer. Therefore, physicians need to pay close attention to the possibility of uterine cancer when a female patient presents clinically with similar cancer symptoms and has a young age of menarche.

This study has the following limitations: 1 this was a cross-sectional study in which there was no temporal relationship and causality cannot be determined; 2 there was a recall bias in the reporting of this questionnaire; and 3 this experiment may have included some confounding factors for failure to participate in surveys. Such as parents' education level, changes in family status, child's birth weight, family history of cancer, genetic factors, etc.; and 4 spanning a long period of time, 16 years ago, individual education level and physical level may have changed.

## Comparison with similar studies

There are similarities between a previously published study and ours, but there are some significant differences between our studies. In a Mendelian randomization study on ovarian cancer, it was noted that age at menarche was associated with an increased risk of ovarian cancer [36]. Not coincidentally, age at menarche was shown to be associated with endometrial cancer risk in another Mendelian randomization study [37]. Compared with others [38, 39], this study aimed at the relationship between age at menarche and the prevalence of malignancies of the uterus and ovaries. We found a negative correlation with the prevalence of uterine cancer, while the relationship with cervical and ovarian cancers was not significant, so we studied more on uterine cancer. On this basis, we conducted a large number of sensitivity analyses, subgroup analyses and cross-validation, which helped us to find the potential high-risk groups more accurately, and made our findings more meaningful in preventive screening.

## Conclusion

The analysis of the NHANES questionnaire data showed that the younger the age of menarche, the higher the risk of uterine cancer in the population. Furthermore, according to our findings, the correlation between the prevalence of uterine cancer and age at menarche was even stronger. These results suggest that public health workers need to pay attention to the early age of menarche when developing screening programmes for malignancies of the uterus and ovaries to develop more appropriate screening programmes.

## Data Availability

No datasets were generated or analysed during the current study.
